# Characteristics and contributory factors for injectable contraceptive usage among women in Kumasi, Ghana

**DOI:** 10.1186/s40834-016-0019-0

**Published:** 2016-05-26

**Authors:** Dennis Odai Laryea, Frank Ankobeah, Emmanuel S. K. Morhe, Yaw Ampem Amoako, Kathryn Spangenberg

**Affiliations:** 1 Public Health Unit, Komfo Anokye Teaching Hospital, Kumasi, Ghana; 2 Department of Obstetrics and Gynaecology, College of Health Sciences, Kwame Nkrumah University of Science and Technology, Kumasi, Ghana; 3 Directorate of Obstetrics and Gynaecology, Komfo Anokye Teaching Hospital, Kumasi, Ghana; 4 Directorate of Medicine, Komfo Anokye Teaching Hospital, Kumasi, Ghana; 5 Directorate of Family Medicine, Komfo Anokye Teaching Hospital, Kumasi, Ghana

**Keywords:** Injectable contraceptives, Family planning, Ghana

## Abstract

**Background:**

Preferred methods of contraception vary from country to country. Family Planning services have been available on a large scale in Ghana since the 1980’s and their use has contributed to gradual decline in the total fertility rate from 6.4 in 1988 to 4.2 in 2014. Since their introduction in Ghana in the early 1990’s, Injectable contraceptives have seen increasing patronage and are currently the most preferred method of contraception. We set out to identify possible factors contributing to the preference for injectable contraceptives among women in Ghana.

**Methods:**

We conducted a descriptive cross-sectional survey of women accessing contraceptive services at the Family Planning Unit of the Komfo Anokye Teaching Hospital in Kumasi, Ghana. Women who reported for the second dose of their injections were eligible to be selected for participation in the study. Informed consent was obtained from eligible participants. Data was collected using a structured questionnaire in January and February 2011. Data captured included age, marital status, highest level of education completed, religion, ethnicity and employment status, previous contraceptive use, sources of contraceptive information and reasons for choosing injectable contraceptives. Quantitative data was entered into a Microsoft Access Database and analysed using Epi Info Version 7.1.4. Qualitative data was analysed thematically.

**Results:**

A total of 247 respondents participated in the study. One hundred and seventy three (70.0 %) were using Depot Medroxyprogesterone Acetate and 74 (30.0 %) were using NorethisteroneEnanthate/Estradiol Valerate. The mean age for women on Depot Medroxyprogesterone Acetate was higher than those on NorethisteroneEnanthate/Estradiol Valerate (*p* < 0.001). The effectiveness of method, recommendation from other users, low incidence of forgetfulness and the relatively longer intervals for administration were the commonest reasons for the use of injectables among respondents. The majority of users, 225 (91.1 %), were satisfied with the method and will recommend it to other potential users. Only 10.8 % of the 68 respondents reporting undesirable effects of the injectables intend to change the method.

**Conclusion:**

A high level of satisfaction exists among current users of injectables in Ghana and is influenced by a variety of factors. Strategies to increase the uptake of injectables can go a long way to increase the contraceptive prevalence rate and reduce the unmet need for Family Planning in Ghana.

## Background

Various methods of contraception are available globally and various factors may influence the use. Although there is no internationally recognized ‘ideal’ method mix, skewed uptake of a contraceptive method may reflect local cultural preferences or social norms, but may become worrisome if it results from restrictive population policies, lack of access to a broad range of methods, or provider bias [1]. The most preferred methods of contraception vary from country to country. In Nigeria, the Intrauterine Contraceptive Device (IUD), male condoms and injectables have been identified as the most patronised methods [2–4] while in Canada, Oral Contraceptives, Condoms and Sterilisation have been found to be the preferred methods [5] with Ethiopia reporting Injectables as the most preferred method [6]. Various reasons underlie the preference of one method over the other [7–11]. Understanding factors that drive contraceptive method choice is important for policy formulation and implementation for the provision of methods that are accessible and acceptable to users [12].

Family Planning services have been available on a large scale in Ghana since the 1980’s and their use has resulted in a decline in Ghana’s fertility rate from 6.4 in 1988 to 4.2 in 2014 [13, 14]. Knowledge on contraception is almost universal in Ghana [13]. Among the various contraceptives available in Ghana are two types of injectables are available, a progestin-only injectable contraceptive, Depot Medroxyprogesterone Acetate (DMPA) given once every three months and a combined-injectable contraceptive (CIC), NorethisteroneEnanthate/Estradiol Valerate (NET-EN/EV) given monthly [15]. Current and prospective users identify injectables as the method of choice [13, 14, 16] but the reasons for these have not been explored. In the present study, we set out to describe the characteristics of women on the two types of injectable contraceptives available in Ghana and to identify possible factors contributing to their preference.

## Methods

This study was conducted at the Family Planning Unit of the Komfo Anokye Teaching Hospital (KATH). KATH is a 1,200-bed tertiary level referral centre providing both basic and specialised services. The Family Planning Unit is part of the Directorate of Obstetrics and Gynaecology. The unit provides a wide array of contraceptive methods including oral contraceptive pills, injectable contraceptives, intrauterine contraceptive devices, male and female condoms and implants. The unit is also equipped with a theatre where minor surgical procedures such as bilateral tubal ligation and vasectomy are performed. Clients accessing services at the unit are mostly from the Kumasi metropolitan area.

This was a descriptive cross sectional study. The study population was women accessing services at the Family Planning Unit of the KATH. Women who reported for the second or subsequent doses of injectable contraceptives were eligible to be recruited into the study. All eligible women were approached for participation in the study over a two-month period, January and February 2011. Potential respondents who agreed to participate following receipt of information on the study were provided with a study information leaflet and informed consent was obtained. Consecutive cases of eligible women who gave consent were included in the study on their respective clinic days. Women on injectables who did not give informed consent or who had participated in the previous month were excluded. Using single population proportion formula for descriptive study (StatCal) a sample size of 250 was determined with following assumptions: 95 % confidence level, 75 % injectable contraceptive users in KATH choose DMPA, confidence limit of 5 %, design effect of 1. This allowed a good representative sample of DMPA and NET-EN/EV users to be included in the study. A structured questionnaire consisting of both open and close-ended questions was employed for data collection. The variables studied included age, marital status, highest level of education completed, religion, ethnicity and employment status, contraceptive methods previously used, sources of contraceptive information, reasons for choosing injectable contraceptives and their experiences using the method. The questionnaire was administered by a trained Research Assistant. Ethical approval for the study was obtained from the KATH/Kwame Nkrumah University of Science and Technology (KNUST) Committee on Human Research, Publications and Ethics.

The data collected was entered into a Microsoft Access database. The quantitative data were analysed using Epi version 7.1.4. Stratified analysis based on the type of injectable used was conducted for all variables. The mean ages for the women on the two different forms of injectable contraceptives were tested for any difference using the Kruskal-Wallis test. The association between the respective type of injectable and specific variables were tested and all computations were done at 95 % CI and a 5 % level of significance. The qualitative data were analysed thematically. Emerging themes were identified and categorised. Subsequently, univariate analysis of the emerging themes was conducted using Epi Info version 7.1.4.

## Results

### Basic demographic information

A total 247 respondents participated in the study. The majority of respondents (70 %) were on DMPA. The ages of respondents ranged from 17 to 52 years with a mean age of 32.8 ± 6.8 years and a median age of 32 years. The age-group 21-40 years accounted for the majority of respondents. More than 50 % of respondents had received only basic education (Primary or JSS/Middle School). Two hundred and twelve respondents (85.8 %) were married (Table 1).

**Table 1:** Demographic information on respondents

	DMPA		NET-EN/EV	
PARAMETER	Frequency	%	Frequency	%
AGE GROUP				
≤20 years	0	0	6	8.1
21-30	59	34.1	36	48.7
31-40	88	50.9	29	39.2
>40	26	15.0	3	4.0
EDUCATION COMPLETED				
No formal education	16	9.3	3	4.0
Primary	37	21.4	13	17.6
JSS/Middle School	86	49.7	40	54.1
Secondary	30	17.3	12	16.2
Tertiary	4	2.3	6	8.1
MARITAL STATUS				
Married	147	85.0	65	87.8
Single	24	13.9	9	12.2
Divorced	2	1.1	0	0.0
RESIDENCE				
Urban	133	76.9	63	85.1
Peri-urban/Rural	40	23.1	11	14.9
ETHNICITY				
Ashanti	131	75.6	53	71.6
Fanti	10	5.9	5	6.7
Ewe	6	3.5	4	5.4
Frafra	6	3.5	1	1.4
Bono	5	2.9	0	0.0
Dagomba	3	1.7	3	4.1
Others	12	6.9	8	10.8
RELIGION				
Christian	165	95.3	65	87.8
Muslim	7	4.1	9	12.2
Other	1	0.6	0	0
OCCUPATION				
Trading	103	59.5	39	52.7
Hairdresser	15	8.7	9	12.2
Unemployed	13	7.5	3	4
Farmer	10	5.8	0	0
Seamstress	8	4.6	8	10.8
Caterer	7	4.1	5	6.8
Teacher	3	1.7	2	2.7
Others	14	8.1	8	10.8
SOURCE OF INFORMATION ON INJECTABLES				
Health worker	61	35.3	31	41.9
Relation/friend	47	27.2	22	29.7
Television	34	19.7	10	13.5
Radio	14	8	7	9.5
Others	17	9.8	4	5.4

The mean age of respondents on DMPA was 33.8 ± 6.8 years with a range of 21 to 52 years. The mean age for respondents on NET-EN/EV was 30.4 ± 6.23 years with an age range of 17 to 42 years. Women on DMPA were older than those on NET-EN/EV (*p* < 0.001; Kruskal-Wallis H = 11.6). Ten (2.8 %) respondents were more than 45 years of age and they were all on DMPA. Education completed and marital status showed no statistically significant relation to the type of injectable used (*p* = 0.10 and 0.60 respectively). Respondents resident in rural/peri-urban did not differ in their choice of injectable contraceptive when compared with urban residents (OR = 1.7; 95 % CI = 0.83- 3.58; *p* = 0.14). Married women accounted for 87.8 % of users of NET-EN/EV compared with 85.0 % of DMPA (Table 2). There was no difference in the choice of injectable when married women were compared with single women (OR = 0.85; 95 % CI 0.37- 1.93; *p* = 0.69). Compared with Muslims, Christians were more likely to use DMPA (OR = 3.26; 95 % CI 1.17- 9.13; *p* = 0.02).

**Table 2:** Injectable type by Marital Status

Marital Status	Type of Injectable	
	DMPA (%)	NET-EN/EV (%)
Married	147 (85.0)	65 (87.8)
Single	24 (13.9)	9 (12.2)
Divorced	2 (1.1)	0 (0.0)
Total	173 (100.0)	74 (100.0)

### Previous use of contraception

A total of 165 (66.8 %) of respondents were using a modern method of contraceptive for the first time. Among those who had previously used a method of contraception, the Combined Oral Contraceptive (COC) was the commonest method accounting for 42.7 %. Other methods previously used were Norplant/Jadelle (15.9 %), IUD (14.6 %), DMPA (12.2 %), NET-EN/EV (6.1 %) with other methods accounting for 9.6 %. Various reasons were assigned for the discontinuation of previous methods of contraception. These were forgetfulness, undesirable side effects (such as amenorrhoea, prolonged bleeding, lower abdominal pain and weight loss).

### Source of information and decision on injectable contraceptives

The leading primary source of information on injectables was health workers, 35.3 % for DMPA and 41.9 % for NET-EN/EV with close relations, friends and the mass media being the other leading sources (Table 1). The decision to opt for an injectable contraceptive among respondents in this study was found to have been based on previous knowledge of the method or recommendation from a friend or relation. A significant proportion of respondents (72 %) also indicated that they had shared information on injectable contraception with relations and/or friends.

### Reasons for use of injectable contraceptives

Information on effectiveness (24.7 %), recommendation from a friend or relation (18.2 %), no associated side effects (14.2 %) and longer intervals between injections (12.9 %) were the leading single most important reasons identified among respondents for opting for injectable contraceptives (Table 3).

**Table 3:** Lead reasons given for opting for injectable contraceptives

Reason	No.	%
Considered as a very effective method of Family Planning/Contraception	61	24.7
Recommendation from a friend/relation	45	18.2
Low incidence/no associated forgetfulness (missed pills)	35	14.2
Relatively longer intervals between injections	32	12.9
Preference for injections	12	4.9
Side effects/dislike of other methods	11	4.5
No specific reason	9	3.6
Desire for regular menses (combined injectable)	8	3.2
Underlying medical condition (told not eligible for other method)	6	2.4
Curiosity	6	2.4
Desire to be discreet (not wanting male partner to know)	4	1.6
Others	18	7.3
Total	247	100.0

Forgetfulness associated with the contraceptive pills accounted for some respondents opting for this method. A 33-year old teacher notes that :

*“With the pill, I sometimes forget but for the injection, I always remember when it is time for my next shot”*

Similar sentiments were expressed by a 25 year old teacher who indicated that:

“*I prefer injections because I do not need to take tablets everyday as I sometimes forget*”

Recommendation from relations was also identified as one of the major reasons respondents opted for injectables. Some of the responses received from participants in this vein include:

*“I chose injections because the one who introduced me to it also uses it”*

*“I was encouraged by the comments a friend made about injections”*

*“I decided to use it because my sister was using the method and testified that it was good”*

*“My sister told me it was a more reliable method compared with pills and I used it because I have a child although not married*”

Some respondents noted that side effects associated with other methods as well as dislike for other methods as reasons for opting for injectables.

*“I don’t have the time to take pills and dislike condoms as well”*

*“I opted for injections because I was having problems with the IUD”*

*“I dislike swallowing tablets”*

Lack of spousal consent and desire to be discreet were also found to be reasons why some opted for this method of contraception. A 26-year old apprentice, a 30-year old trader and another 24-year old trader respectively highlighted their desire to keep the use of contraception from others in the following submissions:

*“I chose injections because I am an apprentice and can take the injection without anyone noticing”*

*“I opted for injectables because my husband did not give his consent for me to take up family planning”*

*“I chose injections so no one knows I am on a family planning method”*

Misinformation about another method of contraception was also identified as a reason one respondent opted for the method. One respondent alluded to this misinformation by other methods saying:

*“I opted for injectables because I had information that the IUD can move from the uterus”*

Some respondents attributed opting for injectable contraceptives to their desire to menstruate regularly. A 30 year old woman on the combined injectable said:

*“I wanted a method that will enable me menstruate monthly”*

### Satisfaction with method

The majority of respondents 225 (91.1 %) expressed satisfaction with the injectable contraceptive currently being used. Among respondents on DMPA, 89.6 % were satisfied compared with 94.6 % of respondents on NET-EN/EV. There was no statistically significant difference (*p* = 0.44) when satisfaction was compared between the two groups (OR = 0.56; 95 % CI = 0.13- 1.79). Among the reasons expressed were effectiveness in preventing pregnancy (31.2 %), ability to space children (23.1 %), peace of mind to focus on other activities (10.1 %) and the low incidence of side effects (4.0 %). Other reported reasons for satisfaction with the method include freedom to decide when to get pregnant, general sense of comfort with reproductive health state and the cessation of heavy menstrual flow. Overall, 95.1 % of respondents in this study indicate that they will recommend the method to someone.

### Side effects of injectable contraceptives

A total of 68 respondents (27.3 %) reported undesirable effects (Tables 4 and 5). Multiple side effects were commoner in women on NET-EN/EV compared with those on DMPA, 21.4 % and 15.0 % respectively. Approximately 55 % of side effects reported among those on DMPA were related to menstruation (irregular menses, a menorrhoea and intermenstrual bleeding). Among respondents who reported undesirable effects, 10.8 % were considering changing their current method.

**Table 4:** Side effects reported by respondents on NET-EN/EV

Side effect	Frequency	%
Multiple	6	21.4
Menorrhagia	5	17.9
Weight gain	4	14.3
Headache	3	10.7
Lower abdominal pain	2	7.1
Others	8	28.6
Total	28	100.0

**Table 5:** Side effects reported by respondents on DMPA

Side effect	Frequency	%
Irregular menses	9	22.5
Prolonged bleeding	7	17.5
Amenorrhoea	6	15.0
Multiple	6	15.0
Headache	3	7.5
Others	9	22.5
Total	40	100.0

## Discussion

Some variations in the characteristics of women on various types of contraceptives exist. Among respondents in this study, a significant proportion were on DMPA (70 %) compared with 30 % on NET-EN/EV and is consistent with reports from the Komfo Anokye Teaching Hospital [16, 17]. Our study also found evidence consistent with data from the Ghana Demographic and Health Surveys (DHS) that married women are more likely to prefer injectables as a method of choice compared with sexually active unmarried women [14]. We found that almost 88 % of women on NET-EN/EV and 85 % of women on DMPA were married women. Married women being in more stable sexual relationships are more likely to prefer relatively longer term contraceptives such as the injectables unlike unmarried women who are less likely to be in a regular sexual relationship hence the observed preference for short term methods of contraception such as male condoms [13]. The higher mean age of respondents on DMPA observed may be due to the fact that women with no desire to return to fertility are more likely to be older and more likely to opt for DMPA because of the relatively longer period for returning to fertility [18]. The preference for NET-EN/EV among relatively younger women may also be due to the perceived predictable return to fertility [19]. The variations observed can be useful in informing policy on contraception in Ghana.

Changing one method of contraception to the other may be influenced by several factors. About a third of respondents had previously used a method of contraception before opting for injectables with the COC being the commonest. Missing COC pills can result in method failure requiring the use of other methods to prevent pregnancy from occurring [20]. Women on injectables are less likely to miss an injection compared with the daily dose of COCs which have been associated compliance challenges and failure of contraception [21–24]. In this study, respondents were not asked about past contraceptive method failures. However, even in the absence of such method failures, the peace of mind offered by injectable contraceptives can be enough reason for some women to opt for this method. The associated forgetfulness and the subsequent need to use other methods may also underlie the high proportion of respondents previously on COCs. Such factors must be stressed on during counselling for family planning in order to reduce any risk of method failure from forgetfulness.

The side effects of methods are important in determining adoption and usage [25] or change of method [11]. Various side effects have been noted for DMPA [26–29] and NET-EN/EV [30]. Sixty-eight (27.3 %) of respondents in this study reported at least one side effect. The low proportion of respondents reporting side effects who are contemplating change of method (10.8 %) indicates a high level of acceptability of injectables and is a likely factor in the high uptake observed in Ghana. Public education on the favourable side effect profile of injectables must be encouraged to ensure an increase in method uptake.

Client satisfaction and the sharing of such experiences in the final decision on the choice of contraception is a very useful observation and can be employed in increasing the contraceptive use in Ghana [13, 14]. A high proportion of users expressed satisfaction with injectables with about 72 % having already shared their pleasant experiences and over 95 % expressing no reservation about recommending them. Injectable contraceptives may continue to be the leading method of choice among Ghanaian women especially the married women if the current trend should continue if highly satisfied clients are used as ambassadors for the method.

Despite the high level of knowledge on contraception in Ghana [13, 14] significant variations exist on knowledge among women on the specific methods of contraception. For example, 91 % of married women have knowledge on injectable contraceptives compared with 80 % for the female condom. The role of knowledge and information in the decision to use a specific method has been demonstrated [2, 9, 31]. Misinformation or inadequate information on contraception can negatively impact the uptake of a method [3, 11, 32]. Ignorance, fear and negative cultural beliefs have been identified as factors likely to prevent the uptake of contraceptives [8, 11].Health workers and relations/friends formed a significant proportion of the primary source of information on contraceptives and they may have also influenced the decision to opt for injectables [10, 33–35]. Health providers as Doctors and Nurses are more likely to give accurate information to clients and therefore less likely to misinform clients although health workers may also impose their choices on clients [10]. Health workers can potentially help translate knowledge on contraception into uptake of methods including injectables.

Although found among a few, the desire to have regular menses was found to be a reason some respondents in our study opted for injectable contraceptive and this is similar to findings in the United Kingdom [7]. NET-EN/EV is associated with regular menses with DMPA known to cause irregular menstrual bleeding and amenorrhoea in some cases. In Ghana, misinformation exists on menstruation. The belief is that failure to menstruate means a woman has dirty blood retained in her and even bleeding for less than 3 to 7 days indicates ill health or causes uterine fibroids [32]. These beliefs may make a method like DMPA less acceptable, because DMPA is generally not associated with regular menses. This study is however unable to establish the role of these beliefs in the desire to menstruate regularly. In contrast, respondents with an extremely busy schedule and no premium on the desire for regular menstruation [7] may find DMPA a more attractive and acceptable option between the two injectable contraceptives.

Ghana’s reproductive health policy is quite liberal. Spousal consent is not a requirement for providing contraception to married women [36, 37]. In our study, some respondents, though a significantly low proportion indicated the reason for opting for the method was to overcome the barrier of spousal consent. Male partner approval has been found to improve the uptake of injectables among married women in the postpartum period [31]. Our study did not consider the timing of the uptake in relation to pregnancy and so is unable to confirm this. It is however clear that spousal consent can influence the uptake of injectables in some cases. The role of providers in this case can also not be ruled out as providers may choose to advise respondents on this method if clients have an expressed desire to be discreet about contraceptive usage in order to maintain marital harmony while meeting their reproductive health needs at the same time.

## Conclusions

The preference for injectables among Ghanaian women is influenced by a myriad of factors. Injectable contraceptives seem well tolerated by current users with a low proportion of current users contemplating discontinuation of the method. The high level of acceptability means injectable contraceptives can contribute to increasing the contraceptive prevalence rate in Ghana and reduce the unmet need for Family Planning.Recent development of subcutaneous injection of Medroxy Progesterone Acetate, to be used by lower level cadres of providers or by self-administration could also be useful in increasing access to contraception in Ghana.

## Abbreviations

COC: Combined Oral Contraceptive
DHS: Demographic and Health Survey
DMPA: depot medroxy-progesterone acetate
IUD: Intrauterine Contraceptive Device
JSS: Junior Secondary School
KATH: Komfo Anokye Teaching Hospital
KNUST: Kwame Nkrumah University of Science and Technology
NET-EN/EV: NorethisteroneEnanthate/Estradiol Valerate
